# Toxic epidermal necrolysis: a paradigm of critical illness

**DOI:** 10.5935/0103-507X.20170075

**Published:** 2017

**Authors:** Alfonso Estrella-Alonso, José Antonio Aramburu, Mercedes Yolanda González-Ruiz, Lucía Cachafeiro, Manuel Sánchez Sánchez, José A. Lorente

**Affiliations:** 1 Instituto de Investigación Sanitaria del Hospital Universitario de Getafe - Madrid, Spain.; 2 Universidad Europea - Madrid, Spain.; 3 Hospital Universitario La Paz-Cantoblanco-Carlos III, Instituto de investigación IdiPaz - Madrid, Spain.; 4 CIBER de Enfermedades Respiratorias - Madrid, Spain.

**Keywords:** Burns, Burn units, Cyclosporin, Immunoglobulins, Stevens-Johnson syndrome, Quemaduras, Unidades de quemados, Ciclosporina, Inmunoglobulinas, Síndrome de Stevens-Johnson

## Abstract

Toxic epidermal necrolysis is an adverse immunological skin reaction secondary in most cases to the administration of a drug. Toxic epidermal necrolysis, Stevens-Johnson syndrome, and multiform exudative erythema are part of the same disease spectrum. The mortality rate from toxic epidermal necrolysis is approximately 30%. The pathophysiology of toxic epidermal necrolysis is similar in many respects to that of superficial skin burns. Mucosal involvement of the ocular and genital epithelium is associated with serious sequelae if the condition is not treated early. It is generally accepted that patients with toxic epidermal necrolysis are better treated in burn units, which are experienced in the management of patients with extensive skin loss. Treatment includes support, elimination, and coverage with biosynthetic derivatives of the skin in affected areas, treatment of mucosal involvement, and specific immunosuppressive treatment. Of the treatments tested, only immunoglobulin G and cyclosporin A are currently used in most centers, even though there is no solid evidence to recommend any specific treatment. The particular aspects of the treatment of this disease include the prevention of sequelae related to the formation of synechiae, eye care to prevent serious sequelae that can lead to blindness, and specific immunosuppressive treatment. Better knowledge of the management principles of toxic epidermal necrolysis will lead to better disease management, higher survival rates, and lower prevalence of sequelae.

## Abstract (es)

La necrolisis epidérmica tóxica es una reacción cutánea adversa de tipo inmunológico secundaria en la mayor parte de los casos a la administración de un fármaco. La necrolisis epidérmica tóxica, el síndrome de Steven Johnson y el eritema exudativo multiforme forman parte del mismo espectro de enfermedad. La mortalidad de la necrolisis epidérmica tóxica es alrededor del 30%. La fisiopatología de la necrolisis epidérmica tóxica es semejante en muchos aspectos a la de las quemaduras dérmicas superficiales. La afectación mucosa del epitelio ocular y genital se asocia con secuelas graves si no se trata de forma temprana. Se acepta en general que los pacientes con necrolisis epidérmica tóxica son tratados mejor en unidades de grandes quemados, donde existe experiencia en el manejo de enfermos con pérdida cutánea extensa. El tratamiento es de soporte, eliminación y cobertura con derivados biosintéticos de la piel de las zonas afectadas, tratamiento de la afectación mucosa, y tratamiento inmunosupresor específico. De los tratamientos ensayados sólo se usa actualmente en la mayor parte de los centros la inmunoglobulina G y la ciclosporina A, aun cuando no existe evidencia sólida para recomendar ningún tratamiento específico. Entre los aspectos particulares del tratamiento de esta enfermedad se encuentra la prevención de secuelas relacionadas con la formación de sinequias, los cuidados oculares para prevenir secuelas graves que pueden conducir a la ceguera, y el tratamiento específico inmunosupresor. Un mejor conocimiento de los principios del manejo de la necrolisis epidérmica tóxica llevará a un mejor manejo de la enfermedad, a una mayor supervivencia y una menor prevalencia de las secuelas.

## INTRODUCTION

Toxic epidermal necrolysis (TEN) is a severe adverse skin reaction consisting of generalized keratinocyte necrosis in the context of inappropriate immune activation by certain drugs or their metabolites. Despite better knowledge of the pathophysiology and important advances in the pharmacological treatment of this disease, mortality remains high. Recent advances related to a better understanding of its pathophysiology and the identification of effective treatments justify the present review. The severity and risk of multi-organ dysfunction of TEN require management by specialists in the critically ill patient with extensive skin loss, such as those who treat burn patients. Therefore, the advances reviewed here are relevant for intensivist physicians. The present narrative review provides an in-depth analysis of the concept, pathogenesis, pathophysiology, and management of TEN.

### Definition, incidence, and epidemiology of toxic epidermal necrolysis

**Figure 1:**
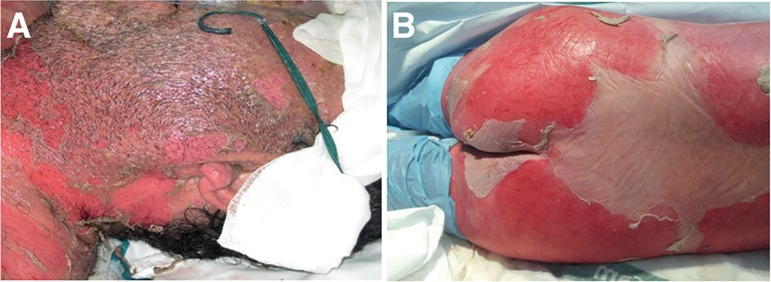
Skin lesions of toxic epidermal necrolysis. Epithelial loss of an extensive surface due to dermo-epidermal detachment is shown.

Toxic epidermal necrolysis is classified within the group of acute blistering diseases (Table 1). It is characterized by inappropriate immune activation in response to certain medications or their metabolites. The separation between the epidermis and the dermis causes blistering and epidermal desquamation (Figure 1). Described by Lyell in 1956^(1)^ as a disease similar to scalds, TEN was initially attributed to staphylococcal infections and medications. Subsequently, it was found that staphylococcal scald and TEN were different entities, with different etiopathogeneses and causes.^(1,2)^

**Table 1:** Classification of exfoliative blistering lesions according to Bastuji-Garin et al.^(3)^

Reaction	Multiform exudative erythema	Stevens-Johnson syndrome	Overlap syndrome	TEN with spots (purpuric erythema)	TEN without spots
Detachment (%)	< 10	< 10	10 - 30	> 30	> 10
Typical lesions	Yes	No	No	No	No
Atypical lesions	Raised	Flat	Flat	Flat	-
Spots	No	Yes	Yes	Yes	No

TEN - toxic epidermal necrolysis.

In 1993, Bastuji-Garin et al.^(3)^ stated that multiform exudative erythema consists of mucosal erosions and patterns characteristic of skin lesions: (a) typical lesions, with concentric "iris" or "target" ring appearance with or without blister formation, and erythematous or purpuric lesions; and (b) atypical lesions, round, reminiscent of papular polymorphous erythema but with only two areas and ill-defined borders, with symmetrical and preferentially acral distribution.

Stevens-Johnson syndrome (SJS) is characterized by mucosal erosions, bullous lesions, and generalized purpuric macules, flat and always symmetrical, often confluent, with a positive Nikolsky sign of detachment. SJS presents with epidermal detachment that affects < 10% of the body surface, whereas the involvement of 10-30% of the body surface defines SSJ/TEN overlap syndrome.

The multiform exudative erythema includes post-infection cases or cases related to drug exposure and has low morbidity and mortality. SJS is a drug-related adverse disorder and presents greater severity and significant mortality. TEN is the most severe form of the disease spectrum and has an incidence of 0.4 to 1.9 cases per million inhabitants per year. The combined total incidence of SJS, overlap syndrome, and TEN is estimated at 2 - 7 cases per million inhabitants per year.^(2,4)^ The conditions are slightly more frequent in women, with a female/male ratio of 1.7.

Multiform exudative erythema, SSJ, TEN, and the intermediate form called overlap syndrome are part of the same disease spectrum (Table 1).

Toxic epidermal necrolysis is associated with immunosuppression states (e.g., bone marrow transplantation), HIV infection, connective tissue diseases, and malignancy (leukemias, lymphomas, and solid tumors).^(5-7)^ In Spain, approximately 50 - 60 cases are diagnosed per year, for an incidence of approximately 0.93 - 1.89 cases per million inhabitants per year.

### Prognosis

In a systematic review, the mortality rate of 708 patients with TEN was 30%. Complicated sepsis with multiple organ failure was the most common cause of death.^(8)^

A score (SCORTEN) was recently developed to assess the severity and to predict the mortality of TEN according to 7 easy-to-measure items^(9)^ and has been validated to estimate mortality on days 1 and 3 of hospitalization.^(10)^ Mortality is related to the value of the score (Table 2).^(9)^ It should be noted that due to advances in the management of TEN, it is possible that the SCORTEN overestimates mortality in centers with experience.

**Table 2:** Prognostic factors of toxic epidermal necrolysis (SCORTEN score)*

**Criteria: 1 point for each condition**
Age > 40 years
Heart rate > 120 beats per minute
Diagnosis of malignancy
Epidermal detachment > 10% of body surface on day 1 of hospitalization
Blood urea nitrogen > 28mg/dL
Glucose > 252mg/dL
Bicarbonate < 20mEq/L
Total score (mortality rate): 0 - 1 (3.2%); 2 (12.2%); 3 (35.5%); 4 (58%, 3%); > 5 (90.0%)

* Adapted from: Bastuji-Garin S, Fouchard N, Bertocchi M, Roujeau JC, Revuz J, Wolkenstein P. SCORTEN: a severity-of-illness score for toxic epidermal necrolysis. J Invest Dermatol. 2000;115(2):149-53.(9)

### Etiology

The cause of TEN is an immune response to exposure to drugs or their metabolites mediated by lymphocytes. Cases have been described after vaccination against measles-mumps-rubella (triple viral),^(11)^ *Mycoplasma pneumoniae* infection,^(12)^ and dengue virus, after reactivation of cytomegalovirus infection, and after the administration of contrast agents. However, the vast majority of cases are related to drug hypersensitivity (Table 3).^(13)^ There are also idiopathic forms, triggered by poisons, or that develop as a manifestation of graft-versus-host disease.^(14)^

**Table 3:** Drugs associated with risk of Stevens-Johnson syndrome/toxic epidermal necrolysis (EuroSCAR study)^(13)^

Confirmed high risk	Low risk	Potential risk (requires more evidence)	Risk not determined
Neviparine	Sertraline	Pantoprazole	Statins
Lamotrigine	Acetic acid	Corticosteroids	Diuretic sulfonamides and antidiabetics
Carbamazepine	NSAIDs	Pyrazolones	B-blockers
Phenytoin	Macrolides	Acetylsalicylic acid	ACE inhibitors
Phenobarbital	Quinolones	Tramadol	Ca2+ channel blockers
Cotrimoxazole and other sulfonamides	Cephalosporins	Nimesulide	Diuretic thiazides
Sulfasalazine	Tetracyclines	Paracetamol	Furosemide
Allopurinol	Aminopenicillins	Ibuprofen	Insulin
Oxicam and other NSAIDs			Propionic acid NSAIDs
			Other proton pump inhibitors
			Other serotonin reuptake inhibitors

NSAIDs - non-steroidal anti-inflammatory drugs; ACE - angiotensin-converting enzyme; Ca2+ - calcium.

The increased risk is largely limited to the first 2 months after starting the new treatment. In approximately 20 - 25% of cases, and likely in an even greater proportion of pediatric cases, a clearly responsible drug is not found.^(3,4)^

### Pathogeny

Toxic epidermal necrolysis consists of necrosis and generalized detachment of the epidermis due to keratinocyte apoptosis induced by an immune mechanism, with a genetic basis in certain ethnic populations. The main inducers of keratinocyte apoptosis are cytotoxic CD8+ T lymphocytes (CTL), together with natural killer (NK) cells. Several cytotoxic proteins and cytokines (such as the soluble Fas ligand [FasL], perforin/granzyme, tumor necrosis factor [TNF] alpha, and the TNF-related apoptosis-inducing ligand (TRAIL) have been proposed as mediators of extensive keratinocyte apoptosis.^(15-17)^ Granulysin, a cytolytic protein found in CTL and NK, plays a key role in pathogenesis. Recently, reactive oxygen species (ROS) formed within keratinocytes have also been implicated. It is believed that intracellular damage by ROS precedes the activation of the pro-apoptotic systems.^(16,17)^

Stevens-Johnson syndrome and TEN have a genetic component. The HLA-B12 antigen phenotype is associated with a higher incidence of TEN. Reaction to sulfonamides is associated with A29, B12, and DR7, whereas reaction to oxicam derivatives is associated with A2 and B2.^(15,16)^

The following mechanisms have been implicated in the pathogenesis of TEN:^(15)^ (a) type IV delayed hypersensitivity reaction, (b) cytotoxicity against keratinocytes mediated by some lymphocytic substance, (c) type II cytotoxic reaction, and (d) non-immunologically mediated necrolysis. These factors, together with a predisposition to infection or a certain genetic susceptibility, are currently considered in the pathogenesis of TEN. It has been suggested that keratinocytes abnormally metabolize the responsible agent, producing a metabolite that binds to the HLA molecule on the cell surface and is recognized by cytotoxic lymphocytes. These lymphocytes migrate into the epidermis, react with keratinocytes, and cause epidermal necrolysis.

The epidermal and dermoepidermal infiltrate corresponds to CD8 T lymphocytes, and the dermal infiltrate to CD4 T lymphocytes. Dendritic lymphoid cells apposed to damaged macrophages and necrotic keratinocytes have been observed. At the point of contact with the latter, the plasma membrane is absent. Aberrant HLA-DR expression in keratinocytes has also been noted, a phenomenon also observed in other inflammatory skin diseases.

### Pathophysiology

The pathophysiology of TEN is explained by (i) extensive skin loss, (ii) systemic inflammatory response, and (iii) mucosal involvement.^(18,19)^

First, extensive skin loss is associated with massive fluid loss. The patient may present with prerenal acute renal failure, electrolyte abnormalities (severe hypernatremia), signs of tissue hypoperfusion (hypotension, hyperlactatemia-acidosis), and shock, requiring aggressive fluid resuscitation (*vide infra*). Extensive skin loss is also associated with loss of the barrier function to infections and an increased risk of infection and sepsis by microorganisms that colonize the skin.

Second, the local inflammatory response is associated with release of cytokines into the circulation and a systemic inflammatory response, characterized by tachycardia, tachypnea, fever, and leukocytosis. This systemic inflammatory response situation, similar to that observed in other conditions in critical patients, is associated with hypermetabolism, immunoparalysis, risk of infection, and risk of sequential organ dysfunction.

Third, the involvement of the oropharyngeal and bronchial mucosa leads to the formation of epithelial remnants, dysphagia, difficulty eliminating secretions, formation of atelectasis, and acute respiratory failure. In this context, the patient may require mechanical ventilation and is therefore at risk of presenting the complications associated with ventilatory support.

### Clinical manifestations

The clinical course characteristic of TEN occurs in three phases: the prodromal period, the necrolysis period, and the reepithelialization period.

### Prodromal period

Skin involvement in TEN is preceded by a prodrome of systemic manifestation that includes fever, cough, runny nose, conjunctivitis, appetite loss, and general malaise. The duration of this phase is typically 48 - 72 hours but may last for weeks. It usually occurs 1-3 weeks after the ingestion or application of the suspected medication. Signs in the mucous membranes (eyes, mouth, nose, and genitals) begin after the prodrome in 90% of cases.^(3,6)^

### Necrolysis period

A painful macular exanthema appears suddenly, with a sensation of pain and burning. Initially, these eruptions are distributed symmetrically on the face and upper part of the trunk, generally avoiding the scalp. The eruption spreads rapidly and reaches its maximum in 4 days, though sometimes in hours. The lesions become confluent, and they become a diffuse erythema that curiously avoids the pressure zones covered in clothes. Along with the generalized dark and erythematous eruption, blisters and phlyctenae appear. In the erythematous areas, the epidermis is detached with minimum friction or digital pressure (Nikolsky sign). The process is more severe in places subject to pressure or trauma, such as the back or buttocks. The epidermal detachment can progress for 5 - 7 days, after which a variable period of re-epithelialization occurs (usually 1 - 3 weeks).^(3,6,20)^

### Mucosal involvement

**Figure 2:**
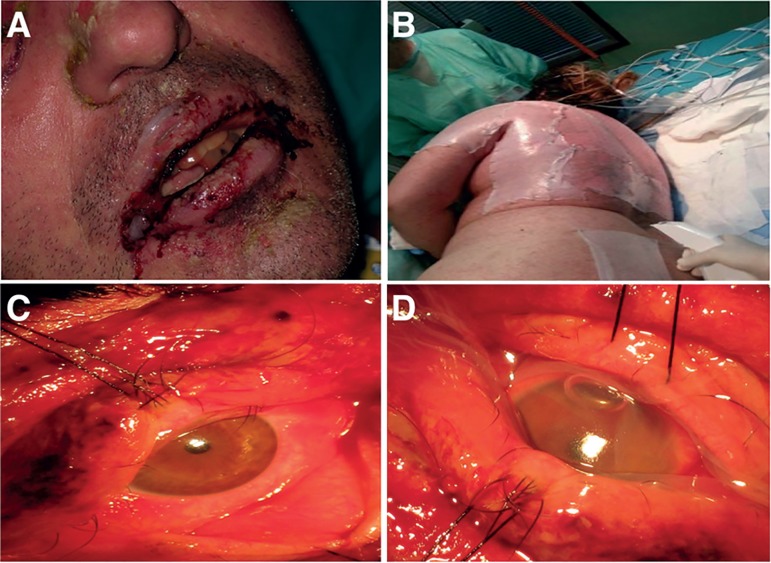
Mucosal involvement in toxic epidermal necrolysis and skin coverage. A) Oral and labial mucosa involvement. B) Skin coverage with Biobrane^®^ dressing. As part of support treatment, coverage of denuded areas reduces fluid and heat loss from the exposed dermis. C) Ocular surface involvement. D) Treatment with amniotic membrane.

Mucosal lesions appear in 90-95% of patients (Figure 2). In one-third of them, mucositis can precede skin lesions by a few days. The mucosal lesions settle, in order of frequency, in the oropharynx, eyes, genitals, and anus, and more rarely in the nose, esophagus, trachea, and bronchi. In more than half of the patients, there is simultaneous involvement of three mucosa, with a single involvement being rare (only 15% of patients). There is no correlation between the severity of the mucosal lesions and the extent of the skin lesions.^(3,20)^

The involvement of the different mucosa leads to the formation of synechiae, with dysfunction and pain, which must be prevented. The patient may present purulent conjunctivitis, mucositis of the mouth and genital area, and complete denudation of the gastrointestinal, respiratory, and genitourinary mucosa. Vulvovaginal involvement or balanoposthitis can lead to urinary retention and vaginal or vaginal canal stenosis.^(3,20)^

Ocular involvement occurs with photophobia, pain, and vision loss and includes keratitis, infection, and permanent vision loss.^(21,22)^

### Re-epithelialization period

The re-epithelialization period lasts between 1 and 3 weeks, depending on the extent and severity of the clinical picture. Hyper- and hypopigmentation occur in virtually all patients. Nails fall off frequently (onychomadesis), and as they grow back, they may develop deformities that are not usually associated with significant functional disability, though they are sometimes lost permanently.

### Histology

**Figure 3:**
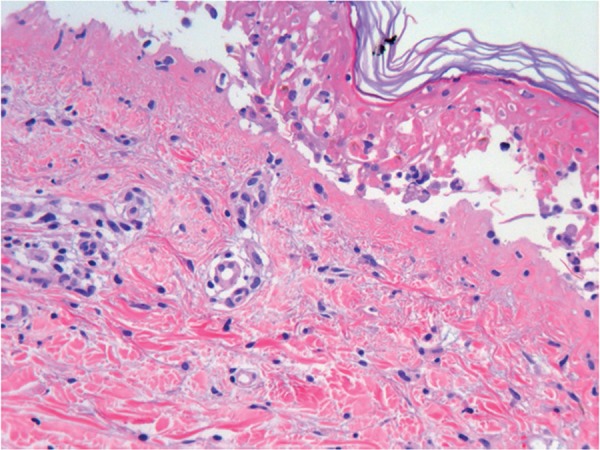
Histology of toxic epidermal necrolysis. Confluent necrosis of epidermal keratinocytes with dermo-epidermal detachment (HE, 120X).

Skin sections affected by TEN show generalized keratinocyte apoptosis and patchy and confluent cell necrosis in the epidermis, separation of the dermo-epidermal junction with formation of subepidermal blisters, and discrete mononuclear infiltrate with a low quantity of eosinophils in the dermis, which corresponds to CTL, some of which are in close contact with necrotic keratinocytes, as observed in graft-versus-host disease (pericellular satellitosis). Skin adnexa may be affected, although less frequently (Figure 3).

### Treatment

Toxic epidermal necrolysis, in the context of skin loss, is associated with systemic changes, and its treatment must be performed in a burn unit by an interdisciplinary team composed of specialists in intensive care, plastic surgery, dermatology, and ophthalmology.^(23,24)^ Studies have documented that survival is greater if patients are transferred early to a burn unit.^(18,23)^ In a retrospective review of 199 patients treated in a burn center, the mortality rate was 32% compared with 51% among patients who were not transferred or who were transferred later.^(23)^

The treatment described here is generally in line with the recently proposed guidelines of the United Kingdom for the management of TEN.^(25)^ Management is based on (i) withdrawal of the causative drug, (ii) support measures (similar to those required for patients with extensive burns), (iii) treatment and prevention of the specific sequelae of SJS/TEN, and (iv) specific systemic treatment of SJS/TEN (immunosuppressive treatment).

### General measures and systemic treatment

The identification and early withdrawal of the aggressor agent as a first measure improves prognosis. Similar to any patient with extensive skin loss from another cause (i.e., burns), the patient must be adequately monitored and must receive appropriate treatment at the burn unit: ensure venous access, consider the need for orotracheal intubation, and monitor the vital signs. The management of the airway may require tracheal intubation in the context of oropharyngeal and upper and lower airway mucosal lesions, causing pain, retention of secretions, and respiratory distress.^(18,19)^

Resuscitation is an aspect of particular importance since the most frequent cause of hemodynamic instability and risk of shock is fluid loss. The criteria for resuscitation are based on volume replacement with crystalloids according to the diuresis of the patient. In complex cases, in which the patient presents cardiorespiratory co-morbidity or shock, invasive hemodynamic monitoring may be necessary.

The support measures are similar to those implemented in the management of burn patients: local care of wounds (following the treatment criteria of superficial burns, which include skin coverage with biosynthetic skin or Biobrane), analgesia, nutritional support, and temperature control (Figure 2). Monitoring of the colonizing flora and early treatment of the infection when there is clinical suspicion is of great importance to prevent sepsis and multiple organ dysfunction.

The patient is managed from the beginning by the intensivist in close collaboration with specialists in plastic surgery, dermatology, ophthalmology, rehabilitation, and psychiatry.

### Treatment and prevention of sequelae

Mucosal involvement can lead to severe acute and chronic complications, such as the development of skin scars, eye lesions, depigmentation, dental complications, genitourinary problems, and lung diseases, the best treatment being the prevention of synechiae formation in different sites.^(24,25)^

Ophthalmological complications develop between approximately 50 to 90% of patients with acute ocular involvement (Table 4).^(21,22)^ A complete ophthalmological examination should be performed, using fluorescein to document epithelial loss, and if present, initiate appropriate treatment to avoid sequelae. The severity of the lesions can be established according to three grades:^(22)^ 0 (without lesions), no ocular involvement; 1 (mild), conjunctival hyperemia; 2 (severe), epithelial defect or pseudomembrane formation; 3 (very severe), presence of both epithelial defect and pseudomembrane formation. The treatment consists of washing with saline to eliminate mucosal remains and inflammatory tissue. In cases with grade 1 severity, corticosteroids and an antibiotic should be applied. Cases with grade 2 or 3 severity should be additionally treated with amniotic membrane transplantation to prevent sequelae and loss of visual acuity. Amniotic membrane transplantation has been shown to be effective in several clinical trials.^(22,26,27)^

**Table 4:** Clinical manifestations and treatment of mucosal involvement and their sequelae^(24)^

Organs/systems	Complication	Management
Tegumentary system	Depigmentation, melanocytic nevus, blistering desquamation, onycholysis, onychodystrophy, and nail and hair thinning and loss	Immediate referral to the specialized unit.
		Elimination of the devitalized epidermis
		Cover with a non-adherent dressing
		Avoid frequent bandage changes that may prevent re-epithelialization
		Biosynthetic silver biological coverage or impregnated antibiotic dressing
		Monitoring of the infection (cultures of the injured skin every 48 hours)
		Use of prophylactic antibiotics is not indicated
		Control of the environmental temperature
		Aseptic handling
		Peripheral venous access away from affected areas
Ocular	Dry eye syndrome, sensation of sand in the eye, symblepharon, corneal scars, trichiasis, blindness, subconjunctival fibrosis, and photophobia	Ophthalmological consultation
		Eye drops every 2 hours
		Lubricants and topical antibiotics
		Avoid development of synechiae by debridement with a blunt instrument
		Transplantation of amniotic membrane if there is involvement of the cornea, conjunctiva or edge of the eyelid
Pulmonary	Bronchitis, bronchiectasis, bronchiolitis obliterans, organizational pneumonia, and respiratory tract obstruction	Monitoring of respiratory function
		Supplemental oxygen if necessary.
		Tracheal intubation and mechanical ventilation if there is airway involvement
		Saline aerosols, bronchodilators, respiratory physiotherapy
Oral cavity	Sicca syndrome and reduced salivary and physiological flow Periodontal disease, gingival inflammation, synechiae, and oral discomfort	Frequent application of antiseptics
		Elimination of oral scabs
Genitourinary	Dysparemia, adhesions, stenosis of the introitus, vulvovaginitis and erosive balanitis, urethral erosions, and genitourinary tract stenosis	Urological and gynecology consultation
		Normal manual lysis to minimize adhesions
		Foley catheter to maintain the permeability of the urinary tract
Gastrointestinal	Esophageal stenosis	Foley catheter to maintain the permeability of the urinary tract
		Monitoring of nutritional status
		Early enteral feeding
		Prevention of stress ulcers

Female genitourinary problems have also been observed, such as dyspareunia, adhesions, and stenosis of the introitus. The aim of treatment is to reduce the formation of adhesions and vaginal adenosis (presence of cervical tissue or metaplastic endometrial granular epithelium in the vulva or vagina). The measures should include administration of intravaginal corticosteroids, use of vaginal molds, and suppression of menstruation. Vaginal antifungal creams can also be used in combination with topical corticosteroids to prevent vaginal candidiasis.^(28)^

Changes in pigmentation and dental complications are also common after TEN (Table 4).^(20,24)^

### Immunosuppressive treatment

The use of corticosteroids in TEN continues to be controversial. Observational studies have shown increases in complications and mortality associated with corticosteroid use.^(29-31)^ Subsequent studies have suggested that if administered early for a short period of time at moderate or high doses (prednisone 1 - 2mg/kg for 3 - 5 days), corticosteroids may be associated with beneficial effects.^(32,33)^ However, a more recent review and meta-analysis of case series has not confirmed any beneficial effect.^(34,35)^

Plasmapheresis has shown beneficial effects in some studies.^(36)^ Its use is based on the principle of the elimination of drugs, their metabolites, and cytotoxic mediators in the blood. However, for studies in which the effect of plasmapheresis was analyzed, the intervention was used in combination with other treatments.

Cyclophosphamide, a potent immunosuppressive agent, is currently out of use in the systemic treatment of TEN.^(37)^ Although it has been reported that its administration is associated with the arrest of disease progression in 24 hours and complete re-epithelialization in 4 - 7 days,^(37)^ the benefit has not been verified, and its administration is associated with serious complications, such as leukopenia with lymphopenia and sepsis, and death due to septic shock.

The intravenous immunoglobulin IgG was initially proposed as a treatment for TEN based on the concept that FasL is the main mediator of keratinocyte apoptosis.^(38)^ The evidence supporting the use of immunoglobulin is limited. After initial experience with low doses of immunoglobulin (1.0 - 1.5g/kg in one dose), subsequent studies administered higher doses (from 2 to more than 4g/kg). In general, it has not been possible to demonstrate a beneficial effect in a review of the published case series^(39)^ in a cohort of patients with SJS/TEN of the EuroSCAR study,^(40)^ in a retrospective series study,^(41)^ or in several systematic reviews or meta-analyses.^(42-45)^

Anti-TNF strategies are attractive alternatives for the treatment of SJS/TEN. Thalidomide, a potent inhibitor of TNF-alpha, was tested in a clinical trial that was prematurely terminated by increased mortality in the treatment group.^(46)^ Infliximab and etanercept have shown benefits in a small number of cases in uncontrolled studies. They have been administered frequently late in the course of the disease and after many other treatments or in combination, which does not allow an adequate assessment of efficacy.^(47,48)^ Therefore, their efficacy has not yet been demonstrated.

N-acetylcysteine is an antioxidant agent and inhibitor of the pro-inflammatory transcription factor NF-kB. Two case series have shown a beneficial response to N-acetylcysteine, but larger studies are clearly needed to determine if this treatment is associated with beneficial effects.^(49,50)^

Cyclosporin A has been shown to be effective in different studies, including a study by the authors, in which a group of patients who received this treatment was compared with a historical control group.^(51)^ The basis of its use is the recognition of the role of granulysin in the apoptosis that occurs as a result of TEN. Several authors described beneficial effects associated with the use of cyclosporine in isolated cases.^(52)^ The dose was 4 mg/kg/day, orally, divided into two doses, lasting no longer than 4 weeks. The objective was to slow down disease progression, with the onset of re-epithelialization in 2-5 days after the start of treatment. Cyclosporine is well tolerated by most patients.^(51)^ The RegiSCAR cohort study also showed a survival benefit for patients treated with cyclosporine and anti-TNF agents.^(34)^ In our study,^(51)^ we observed better outcomes in 10 patients treated with cyclosporine compared with 6 patients treated with cyclophosphamide and corticosteroids, including shorter re-epithelialization time and lower mortality. In another retrospective study, only 1 of 15 patients treated with cyclosporine died, compared with 2.4 expected deaths based on the SCORTEN score.^(41)^ A recent meta-analysis supports the efficacy of cyclosporine in the treatment of TEN.^(53)^

## CONCLUSION

In summary, toxic epidermal necrolysis is a serious disease that must be treated in burn centers, where experience in the management of the complications of extensive skin loss ensures the best results. The pathophysiology of the condition (fluid loss, risk of multiple organ dysfunction, risk of sepsis) is common to that in patients suffering extensive burns. There is no robust evidence to recommend a specific pharmacological treatment. In general, treatments with corticosteroids and cyclophosphamide are currently in disuse; different centers use immunosuppressant treatment strategies, such as immunoglobulin or cyclosporin A. The value of double or multimodal pharmacotherapy is unknown.
